# Maternal Tn Immunization Attenuates Hyperoxia-Induced Lung Injury in Neonatal Rats Through Suppression of Oxidative Stress and Inflammation

**DOI:** 10.3389/fimmu.2019.00681

**Published:** 2019-04-04

**Authors:** Chung-Ming Chen, Jaulang Hwang, Hsiu-Chu Chou

**Affiliations:** ^1^Department of Pediatrics, Taipei Medical University Hospital, Taipei, Taiwan; ^2^Department of Pediatrics, School of Medicine, College of Medicine, Taipei Medical University, Taipei, Taiwan; ^3^Taipei Cancer Center, Taipei Medical University, Taipei, Taiwan; ^4^Department of Anatomy and Cell Biology, School of Medicine, College of Medicine, Taipei Medical University, Taipei, Taiwan

**Keywords:** hyperoxia, vaccine, interleukin-4, 8-hydroxy-2′-deoxyguanosine, mean linear intercept, von Willebrand factor

## Abstract

Hyperoxia therapy is often required to treat newborns with respiratory disorders. Prolonged hyperoxia exposure increases oxidative stress and arrests alveolar development in newborn rats. Tn antigen is N-acetylgalactosamine residue that is one of the most remarkable tumor-associated carbohydrate antigens. Tn immunization increases the serum anti-Tn antibody titers and attenuates hyperoxia-induced lung injury in adult mice. We hypothesized that maternal Tn immunizations would attenuate hyperoxia-induced lung injury through the suppression of oxidative stress in neonatal rats. Female Sprague–Dawley rats (6 weeks old) were intraperitoneally immunized five times with Tn (50 μg/dose) or carrier protein at biweekly intervals on 8, 6, 4, 2, and 0 weeks before the day of delivery. The pups were reared in room air (RA) or 2 weeks of 85% O_2_, creating the four study groups: carrier protein + RA, Tn vaccine + RA, carrier protein + O_2_, and Tn vaccine + O_2_. The lungs were excised for oxidative stress, cytokine, vascular endothelial growth factor (VEGF) and platelet-derived growth factor (PDGF) expression, and histological analysis on postnatal day 14. Blood was withdrawn from dams and rat pups to check anti-Tn antibody using western blot. We observed that neonatal hyperoxia exposure reduced the body weight, increased 8-hydroxy-2-deoxyguanosine (8-OHdG) expression and lung cytokine (interleukin-4), increased mean linear intercept (MLI) values, and decreased vascular density and VEGF and PDGF-B expressions. By contrast, Tn immunization increased maternal and neonatal serum anti-Tn antibody titers on postnatal day 14, reduced MLI, and increased vascular density and VEGF and PDGF-B expressions to normoxic levels. Furthermore, the alleviation of lung injury was accompanied by a reduction in lung cytokine and 8-OHdG expression. Therefore, we propose that maternal Tn immunization attenuates hyperoxia-induced lung injury in neonatal rats through the suppression of oxidative stress and inflammation.

## Introduction

Respiratory distress syndrome is a major cause of morbidity and mortality in preterm neonates (1). Hyperoxia therapy is often required to treat newborns with respiratory disorders. However, supplemental oxygen administered to newborn infants with respiratory failure increases oxidant stress and leads to lung injury. The rat model is appropriate to study the effects of hyperoxia on preterm infants with respiratory distress because rats are born at the saccular stage, equivalent to an ~30 week human gestation (2). Prolonged exposure of neonatal rodents to hyperoxia resulted in decreased alveolar septation and increased terminal air space size, similar to human bronchopulmonary dysplasia (3, 4). Despite early surfactant therapy, optimal ventilation strategies, and increased use of noninvasive positive pressure ventilation, bronchopulmonary dysplasia remains a major cause of morbidity and mortality during the first year of life, and many infants experience significant respiratory morbidity, including decreased response to acute hypoxia, increased airway reactivity, and development of obstructive airway disease throughout childhood (5–7). No effective clinical therapy is currently available to prevent the development and long-term pulmonary sequelae of bronchopulmonary dysplasia.

Tn antigen is N-acetylgalactosamine residue that is α-linked to a serine or threonine residue, which is one of the most remarkable tumor-associated carbohydrate antigens, often offered to mucin-type carbohydrates (8). Studies have demonstrated that inflammatory cytokines can promote glycan epitope by regulating specific glycosyltransferases (9, 10). Using the linear array epitope technology, Chiang et al. developed an anti-Tn vaccine that induces anti-Tn antibodies with high specificity and high affinity in mice (11). These results suggest that Tn may show immunogenicity and protection in preclinical animal studies. Tn immunization attenuates hyperoxia-induced lung injury in adult mice by inhibiting the nuclear factor-kappa B (NF-κB) activity (12). The effects of Tn immunization on neonatal hyperoxia-induced lung injury are unknown. Therefore, we hypothesize that the maternal Tn immunizations would attenuate hyperoxia-induced lung injury in neonatal rats. This study investigated the protective effects and mechanisms of Tn immunization on lung inflammation and development in neonatal rats exposed to hyperoxia.

## Materials and Methods

### Tn Vaccine Preparation

Tn vaccine was prepared by conjugating Tn to a novel carrier protein as described previously (11). Tn was conjugated to mFc(Cys42)Histag2 or GST(Cys6)Histag2 at a glycotope–carrier protein weight ratio of 5:1. The conjugation was performed in a buffer containing 20 mM sodium phosphate, pH 7.9, 8 M urea, 500 mM imidazole, and 0.2 mM tris(2-carboxyethyl) phosphine (TCEP). After 48 h, the conjugate was refolded in phosphate-buffered saline (PBS) with 0.2 mM TCEP. GST(Cys6) was dialyzed against PBS with 0.2 mM TCEP. Different glycotopes and a linker (N-succinimidyl-6-maleimidocaproate) were conjugated to GST(Cys6) at 4°C for 48 h.

### Animal Model and Experimental Groups

Female Sprague–Dawley rats (6 weeks old) were obtained from BioLASCO Taiwan Co., Ltd and were housed in individual cages with 12-h light–dark cycles. Laboratory food and water were available *ad libitum*. The female rats were randomly assigned to the Tn immunization or control treatment groups (Supplement Figure 1). The Tn immunization strategy consisted of an intraperitoneal injection of Tn (50 μg/dose) in 0.5 mL normal saline, and the control immunization consisted of the intraperitoneal injection of the same volume of carrier protein. The immunizations were administered five times at biweekly intervals on 8, 6, 4, 2, and 0 weeks before the day of delivery. Female rats in estrus or proestrus were placed in cages with adult male rats (two females for each male) for 12 h. The following morning, mating was confirmed by the presence of a vaginal plug, which was considered day 0 of gestation. The dams were allowed to deliver vaginally at term. Within 12 h of birth, litters were pooled and randomly redistributed to the newly delivered mothers, and the pups were then randomly assigned to room air (RA) or oxygen-enriched atmosphere (O_2_) treatment. The pups in the O_2_ treatment subgroups were reared in an atmosphere containing 85% O_2_ from postnatal days 1 to 14. The pups in the control subgroups were reared in normobaric RA for 14 days. Four study groups were obtained as follows: carrier protein + RA, Tn vaccine + RA, carrier protein + O_2_, and Tn vaccine + O_2_. To avoid oxygen toxicity in the nursing mothers, they were rotated between the O_2_ treatment and RA control litters every 24 h. An oxygen-rich atmosphere was maintained in a transparent 40 × 50 × 60-cm plexiglass chamber receiving O_2_ continuously at 4 L/min. Oxygen levels were monitored using a ProOx P110 monitor (NexBiOxy, Hsinchu, Taiwan). On postnatal day 14, pups from each group were deeply anesthetized with an overdose of isoflurane, and body and lung weights were noted. Blood was withdrawn from dams and rat pups to check anti-Tn antibody using western blot. The study protocol was approved by the Institutional Animal Care and Use Committee of Taipei Medical University.

### Western Blot Analysis of the Serum Anti-Tn Antibody

Solubilized proteins were separated using SDS-PAGE and were electrophoretically transferred to polyvinylidene difluoride (PVDF) membranes. PVDF membranes were rinsed in TBS buffer and blocked for 1 h with TBS buffer containing 5% skim milk. After washing with TBST (Tris-buffered saline, 0.1% Tween 20), the PVDF membranes were incubated overnight with rat serum (1:1000) dissolved in antibody buffer. After multiple washing with TBST, the membranes were incubated for 45 min with Jackson AffiniPure Donkey AntiRat IgG (H+L) (1:5000, Jackson ImmunoResearch Laboratories, Inc., West Grove, PA, USA). Membranes were washed, and immunoreactive proteins were detected using Immun-Star assay kit (Bio-Rad) following the manufacturer's suggestions.

### Lung Histology

To standardize analysis, sections were obtained from the right middle lobe of the right lung. The lung tissue was immersed with 4% paraformaldehyde in 0.1 M phosphate buffer (pH 7.4) at 4°C for 24 h. The tissues were then dehydrated in alcohol, cleared in xylene, and embedded in paraffin. Five-micrometer sections were stained with hematoxylin and eosin, examined using light microscopy, and assessed for lung morphometry. Mean linear intercept (MLI), an indicator of mean alveolar diameter, was assessed in 10 nonoverlapping fields (13). Vascular density was determined with von Willebrand factor (vWF) immunohistochemistry reaction (see below).

### Immunohistochemistry of 8-Hydroxy-2′-Deoxyguanosine, von Willebrand Factor, Vascular Endothelial Growth Factor, Platelet-Derived Growth Factor-B, Inducible Nitric Oxide Synthase, and YM-1

Immunohistochemical staining was performed on 5-μm paraffin sections using immunoperoxidase visualization. After routine deparaffinization, heat-induced epitope retrieval was performed by immersing the slides in 0.01 M sodium citrate buffer (pH 6.0). To block the endogenous peroxidase activity and nonspecific binding of antibodies, the sections were preincubated for 1 h at room temperature in 0.1 M PBS containing 10% normal goat serum and 0.3% H_2_O_2_. The sections were then incubated for 20 h at 4°C with mouse monoclonal anti-8-hydroxy-2′-deoxyguanosine (8-OHdG) antibody (1:100; Abcam Inc., Cambridge, MA, USA), rabbit polyclonal antivWF antibody (1:100; Abcam), rabbit polyclonal antivascular endothelial growth factor (VEGF) antibody (1:50; Santa Cruz Biotechnology, Inc., CA, USA), rabbit polyclonal antiplatelet-derived growth factor (PDGF)-B antibody (1:50; Santa Cruz Biotechnology, Inc.), rabbit polyclonal anti-inducible nitric oxide synthase (iNOS) (1:100; Thermo Fisher Scientific, Rackford, IL, USA), or rabbit polyclonal anti-Ym-1 (1:25; STEMCELL Technologies Inc., Vancouver, Canada) as primary antibodies. The sections were then treated for 1 h at 37°C with biotinylated goat anti-mouse or rabbit IgG (1:200, Jackson ImmunoResesarch Laboratories Inc., PA, USA). Following the reaction produced using reagents from an avidin–biotin complex kit (Vector Laboratories, Inc., CA, USA), the reaction products were visualized using a diaminobenzidine substrate kit (Vector Laboratories, Inc.) according to the recommendations of the manufacturer. The 8-OHdG staining was quantified by considering the positively stained nuclei per high-power field. Positively stained cells were counted in five fields randomly selected from each section using a light microscope (magnification: × 400), and results were expressed as the percentage of positively stained nuclei to total cells. Microvessel density was determined by counting the vessels with the positive vWF stained in an unbiased manner and a minimum of four random lung fields at × 400 magnifications (14). The automatic object counting and measuring process was used to quantify the immunoreactivity in the sections. This generated a percentage of positively stained cells, and the values were expressed as a labeling index (%). The positive immunostaining of lung parenchyma for iNOS and Ym-1 were measured at ×400 magnification by the density of immunostained chromogen (0.1 mm^2^) using the Image Pro Plus (Media Cybernetics, Silver Spring, USA).

### Cytokine Assay

Approximately 100 mg of lung tissue from each pup was homogenized, sonicated, and centrifuged at 500 × g for 20 min at 4°C to remove cellular debris according to the manufacturer's instructions. The levels of interleukin-4 (IL-4) in the supernatants were determined using the enzyme-linked immunosorbent assay kit (MyBioSource, San Diego, CA, USA) as IL-4 expression was significantly increased in the lungs from hyperoxia-exposed neonatal rats and mice (15, 16).

### Western Blot Analysis of Growth Factors

Lung tissues were homogenized in ice-cold buffer containing 50 mM Tris·HCl (pH 7.5), 1 mM EGTA, 1 mM EDTA, and protease inhibitor cocktail (complete minitablets; Roche, Mannheim, Germany). The samples were sonicated and then centrifuged at 500 *g* for 20 min at 4°C to remove cellular debris. Proteins (30 μg) were resolved on 12% SDS-PAGE under reducing conditions and electroblotted to a PVDF membrane (Immobilon^P^, Millipore, Bedford, MA, USA). After blocking with 5% nonfat dry milk, the membranes were incubated with antibody against VEGF (1:1000; Santa Cruz Biotechnology, Inc.), PDGF-B (1:1000; Santa Cruz Biotechnology, Inc.), or anti-β-actin (1:20,000; Sigma-Aldrich, St. Louis, MO, USA) and subsequently with horseradish peroxidase-conjugated goat anti-rabbit IgG or anti-mouse IgG (Pierce Biotechnology, Rockford, USA). Protein bands were detected using SuperSignal Substrate from Pierce. Densitometric analysis was performed to measure the intensity of VEGF, PDGF-B, and β-actin bands using AIDA software.

### Statistical Analysis

All data were presented as mean ± SD. Statistical analyses were performed using a two-way analysis of variance with a Bonferroni *post hoc* test for multiple group comparisons. The survival rate was evaluated using the Kaplan–Meier method, and log-rank test was used for intergroup comparisons. Differences were considered statistically significant when *p* < 0.05.

## Results

Three Tn immunization-treated and three carrier protein-treated female rats were successfully mated with male rats. Six dams gave birth to a total of 46 pups; 23 pups each were randomly distributed to the RA and hyperoxia groups. A total of 11 and 12 pups received carrier protein and Tn immunization in the RA groups, and 11 and 12 pups received carrier protein and Tn immunization in the hyperoxia groups.

### Western Blot Analysis of Serum Anti-Tn Antibody

**Figure 1:**
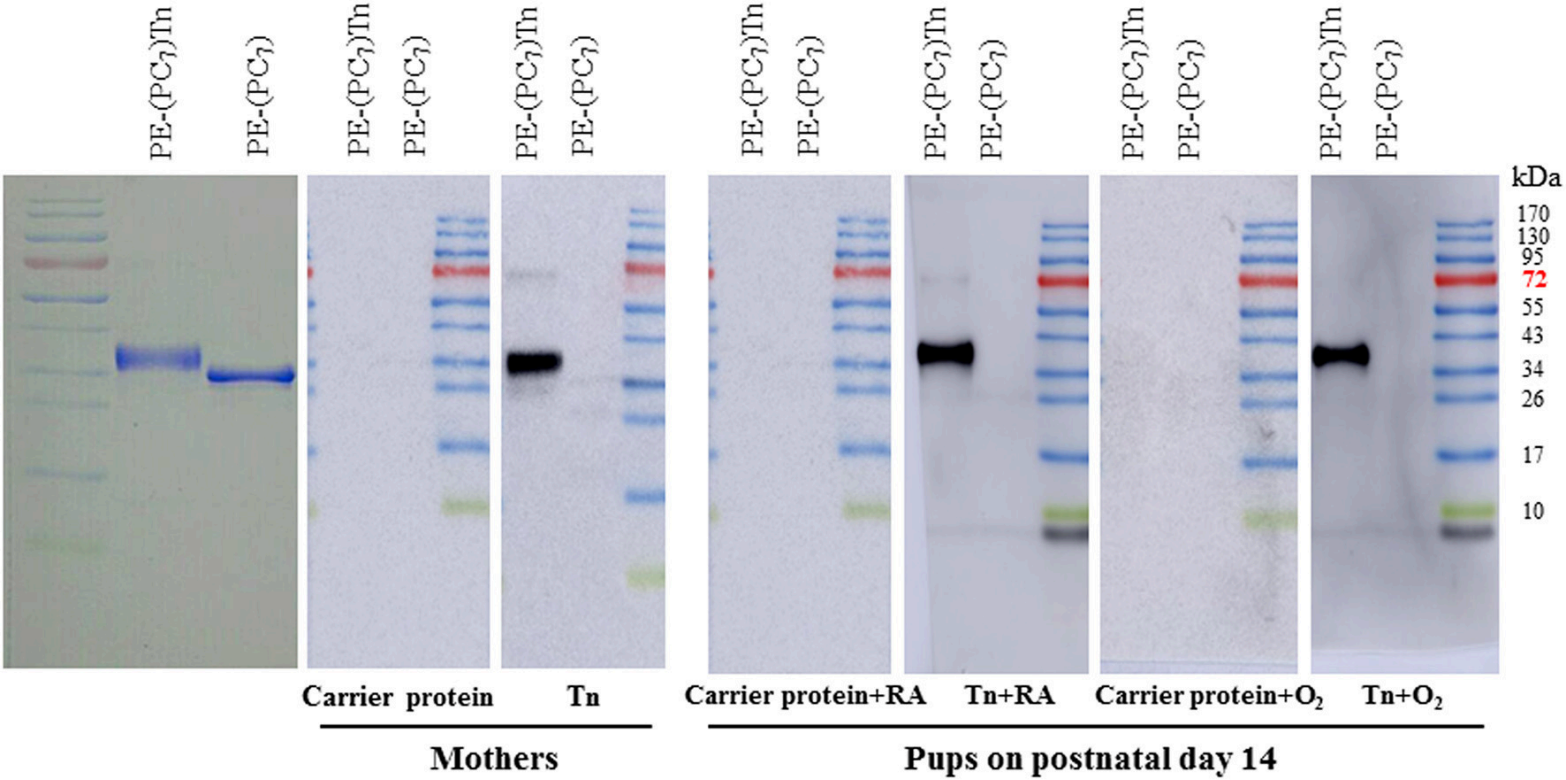
Western blot analysis of serum anti-Tn antibody in dams and rat pups on postnatal day 14. PE-(PC_7_)Tn predominantly recognized one major band (anti-Tn antibody), which was not recognized by PE-(PC_7_). Mothers and pups who received Tn immunization exhibited a dense anti-Tn antibody band, whereas mothers and pups who received carrier protein immunization did not exhibit anti-Tn antibody. The diagram illustrates representative data from three experiments.

PE-(PC_7_)Tn recognized predominantly one major band (anti-Tn antibody), which was not recognized by PE-(PC_7_) (Figure 1). Mothers and pups receiving Tn immunization exhibited a dense anti-Tn antibody band, whereas mothers and pups receiving carrier protein immunization did not exhibit anti-Tn antibody.

### Survival

**Figure 2:**
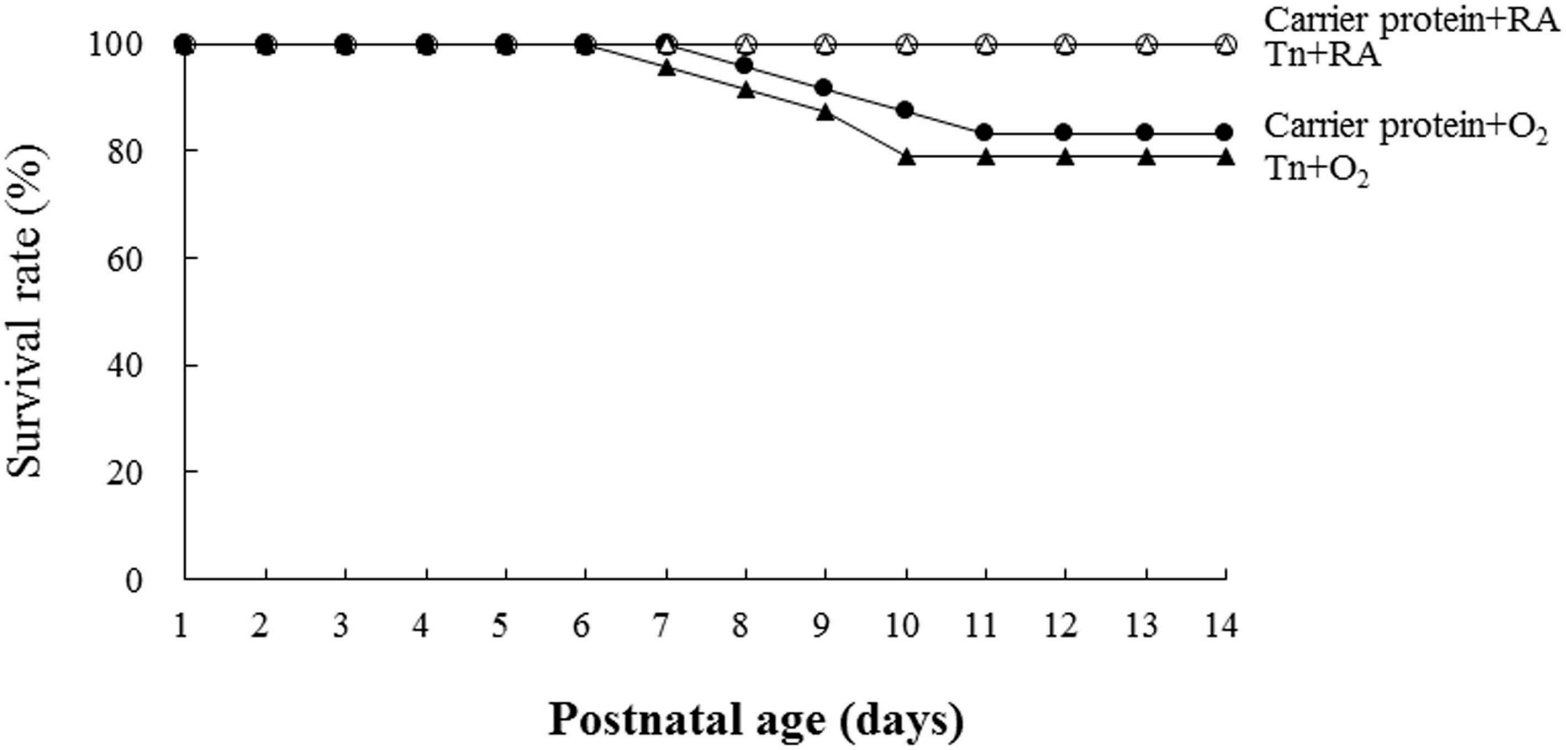
Effects of Tn immunization on the survival rate on postnatal day 14. The rats in the carrier protein + RA or Tn vaccine + RA group all survived. The rats in the carrier protein + O_2_ or Tn vaccine + O_2_ group exhibited a lower survival rate after postnatal day 7. On postnatal day 14, the survival rate between the rats treated with carrier protein or Tn immunization were comparable.

The rats reared in the carrier protein + RA or Tn vaccine + RA group all survived (Figure 2). The rats reared in the carrier protein + O_2_ or Tn vaccine + O_2_ group exhibited a lower survival rate after postnatal day 7. On postnatal day 14, the survival rate between the rats treated with the carrier protein or Tn immunization were comparable.

### Body Weight, Lung Weight, and Lung-To-Body Weight Ratios

The rats in the carrier protein + O_2_ or Tn vaccine + O_2_ group exhibited significantly lower body and lung weights on postnatal day 14 than those reared in the carrier protein + RA or Tn vaccine + RA group (Table 1). Maternal Tn immunization increased the body weight on postnatal day 14 in rats reared in RA or hyperoxia. The rats in the carrier protein + O_2_ group exhibited a significantly higher lung-to-body weight ratio than those in the other three groups.

**Table 1:** Body weights, lung weights, and lung-to-body weight ratios of rat pups on postnatal day 14.

**Treatment**	***n***	**Body weight (g)**	**Lung weight (g)**	**Lung-to-body weight ratio (%)**
Carrier protein + RA	11	20.36 ± 1.43	0.33 ± 0.02	1.60 ± 0.12
Carrier protein + O_2_	9	15.11 ± 1.58^a^	0.31 ± 0.05	2.08 ± 0.48^c^
Tn vaccine + RA	12	23.67 ± 2.12	0.36 ± 0.02	1.55 ± 0.11
Tn vaccine + O_2_	10	19.35 ± 0.82^a^, ^b^	0.33 ± 0.02	1.71 ± 0.11

*Values are mean ± SD*.

a *p < 0.001 vs. carrier protein + RA and Tn vaccine + RA*.

b *p < 0.001 vs. carrier protein + O_2_*.

c *p < 0.05 vs. carrier protein + RA, Tn vaccine + RA, and Tn vaccine + O_2_*.

### Immunohistochemistry for 8-OHdG

**Figure 3:**
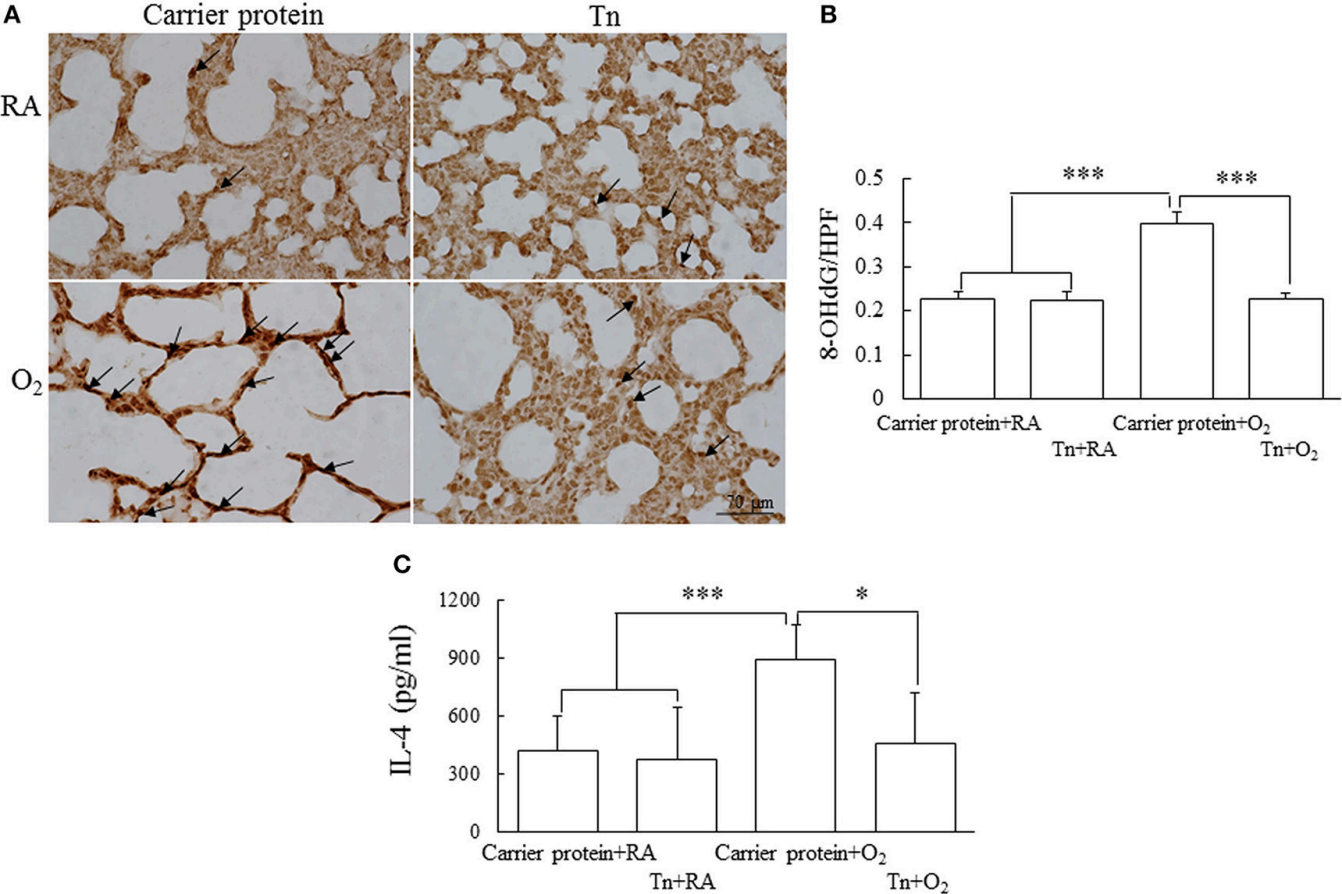
**(A)** Representative immunohistochemistry of 8-hydroxy-2′-deoxyguanosine (8-OHdG), **(B)** quantitative analysis of 8-OHdG-positive cells, and **(C)** lung IL-4 in 14-day-old rats in the carrier protein + RA, Tn vaccine + RA, carrier protein + O_2_, or Tn vaccine + O_2_ group. Positive staining is indicated in brown (arrow). The rats in the carrier protein + O_2_ group exhibited a significantly higher number of positive 8-OHdG cells and IL-4 levels than those in the carrier protein + RA or Tn vaccine + RA group. Maternal Tn immunization significantly decreased the hyperoxia-induced increase in the number of positive 8-OHdG cells and IL-4 levels. ^*^p < 0.05, ^***^p < 0.001.

To investigate whether maternal Tn immunization reduced oxidative stress in neonatal hyperoxia-exposed rat lungs, we used immunohistochemical assays for oxidative stress marker 8-OHdG. The 8-OHdG immunoreactivity was primarily detected in the epithelial cells (Figure 3A). The rats in the carrier protein + O_2_ group exhibited significantly higher number of positive 8-OHdG cells than those in the carrier protein + RA or Tn vaccine + RA group. Maternal Tn immunization significantly decreased the neonatal hyperoxia-induced increase in the number of positive 8-OHdG cells (Figure 3B).

### Cytokine Level

The rats in the carrier protein + O_2_ group exhibited a significantly higher lung IL-4 level than those in the carrier protein + RA or Tn vaccine + RA group (Figure 3C). Maternal Tn immunization significantly decreased the neonatal hyperoxia-induced increase in the lung IL-4 levels.

### Histology Results

**Figure 4:**
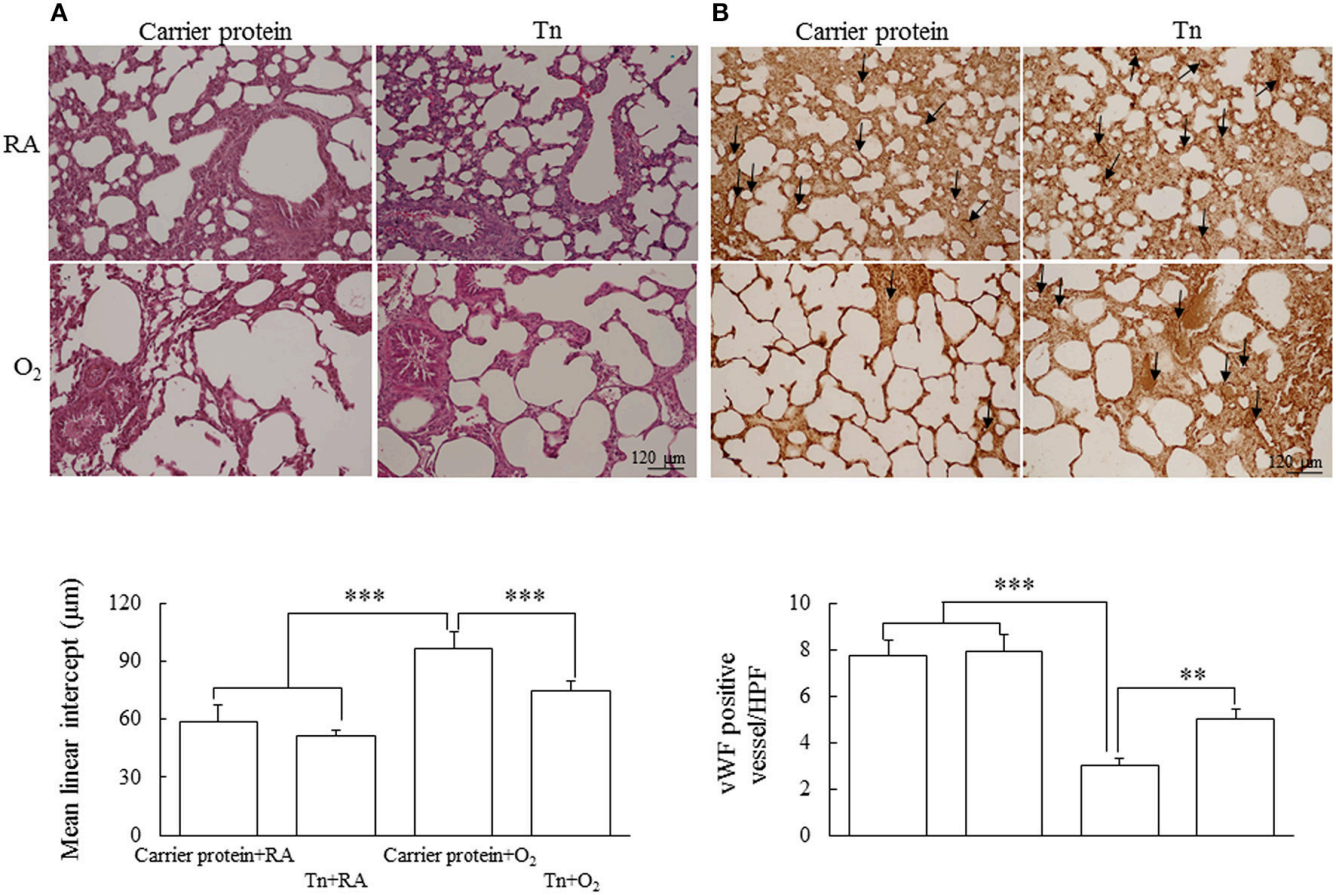
Representative H&E stained lung sections for histology observation and **(A)** mean linear intercept assessment and **(B)** immunohistochemistry of vWF and semiquantitative analysis for vascular density in lung of 14-day-old rats in the carrier protein + RA, Tn vaccine + RA, carrier protein + O_2_, or Tn vaccine + O_2_ group. The rats in the carrier protein + O_2_ group exhibited a significantly higher MLI and lower vascular density than those in the carrier protein + RA or Tn vaccine + RA group. Maternal Tn immunization reversed the MLI and vascular density to normoxic levels. ^**^p < 0.01, ^***^p < 0.001.

Representative lung sections stained with hematoxylin and eosin and vWF from maternal Tn immunization and postnatal hyperoxia-exposed rats on postnatal day 14 are shown in Figures 4A,B, respectively. The rats in the carrier protein + O_2_ group exhibited a significantly higher MLI and lower vascular density than those in the carrier protein + RA or Tn vaccine + RA group. Maternal Tn immunization improved the hyperoxia-induced alteration in the MLI and vascular density to normoxic levels.

### Immunohistochemistry and Western Blotting of VEGF and PDGF-B

**Figure 5:**
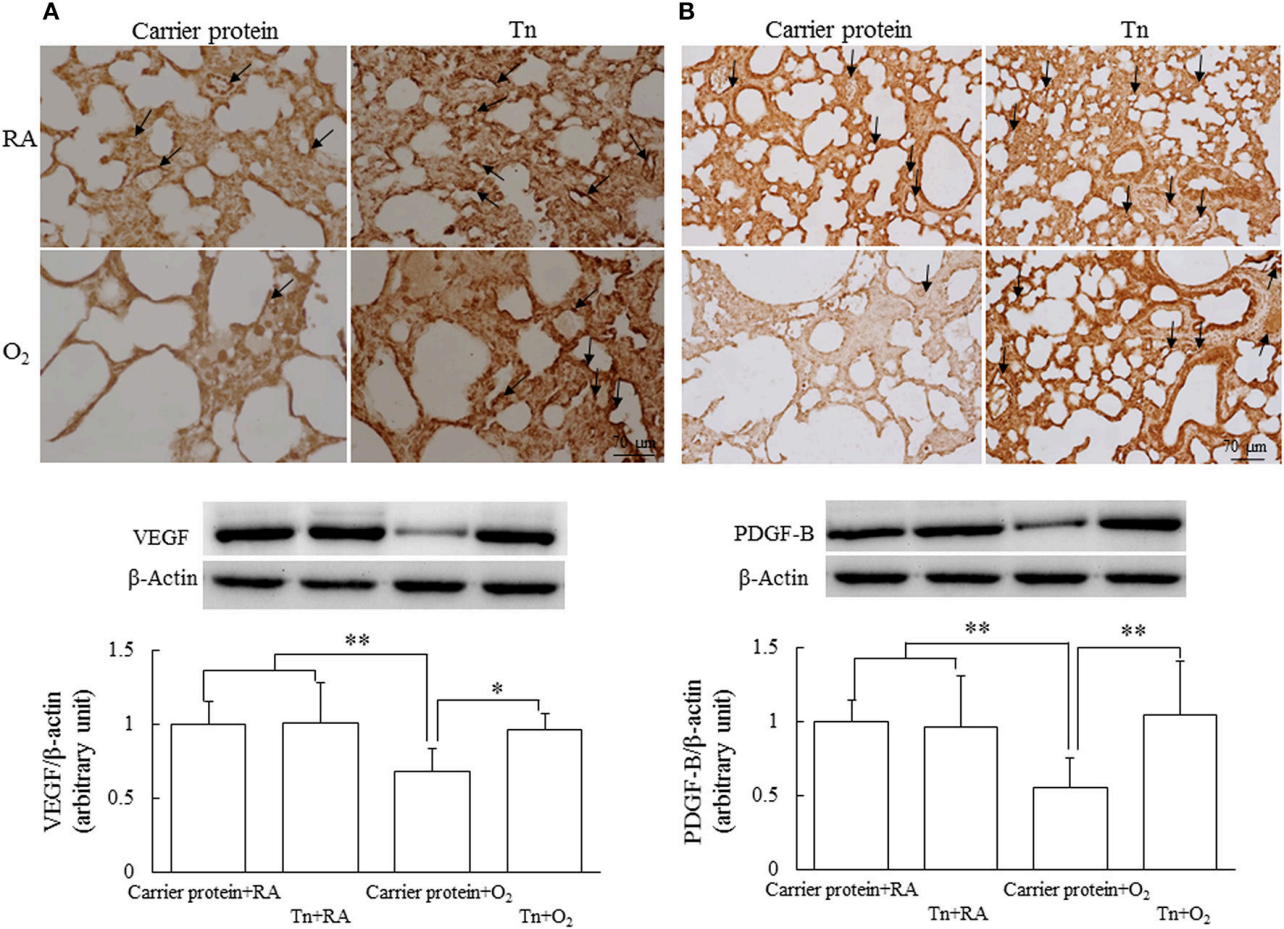
Representative immunohistochemistry and representative western blots and quantitative data for **(A)** VEGF and **(B)** PDGF-B protein expression in 14-day-old rats in the carrier protein + RA, Tn vaccine + RA, carrier protein + O_2_, or Tn vaccine + O_2_ group. The rats in the carrier protein + O_2_ group exhibited significantly lower vascular density and VEGF and PFGF-B expression than those in the carrier protein + RA or Tn vaccine + RA group. Maternal Tn immunization in hyperoxia-exposed rats improved vascular density and VEGF and PDGF-B expression to normoxic levels. ^*^p < 0.05, ^**^p < 0.01.

Representative immunohistochemistry of VEGF and PDGF-B are shown in Figures 5A,B, respectively. The VEGF and PDGF-B immunoreactivities were primarily detected in the endothelial and epithelial cells. The rats in the carrier protein + O_2_ group exhibited significantly lower VEGF and PDGF-B protein expression than those in the carrier protein + RA or Tn vaccine + RA group. Maternal Tn immunization significantly increased the hyperoxia-induced decrease in the VEGF and PDGF-B protein expression.

### M1/M2 Phenotype in Macrophages

**Figure 6:**
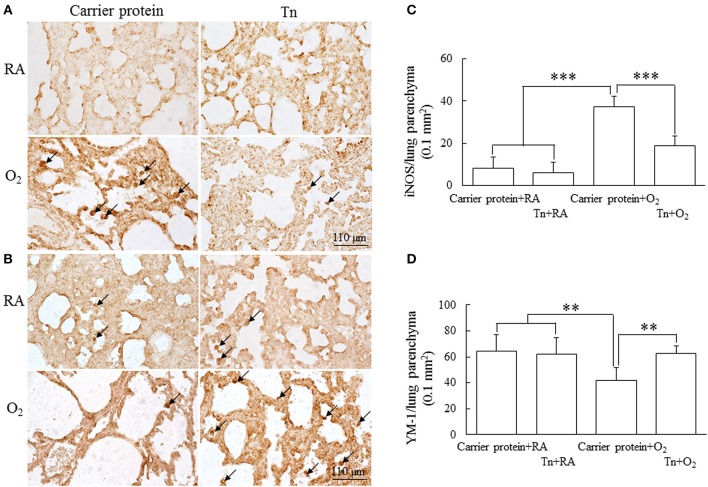
**(A,B)** Representative immunohistochemistry and **(C,D)** quantitative analysis of iNOS and Ym1 positive cells in 14-day-old rats in the carrier protein + RA, Tn vaccine + RA, carrier protein + O_2_, or Tn vaccine + O_2_ group. The rats in the carrier protein + O_2_ group exhibited a significantly higher M1 phenotype (iNOS) macrophages (arrow) and lower M2 phenotype (Ym-1) macrophages than those in the carrier protein + RA or Tn vaccine + RA group. Maternal Tn immunization reversed the hyperoxia-induced M1/M2 macrophage polarization to normoxia levels. ^**^p < 0.01, ^***^p < 0.001.

Representative lung sections stained with iNOS (M1 macrophage maker) and Ym1 (M2 macrophage maker) from maternal Tn immunization and postnatal hyperoxia-exposed rats on postnatal day 14 are shown in Figures 6A,B, respectively. The rats in the carrier protein + O_2_ group exhibited a significantly higher M1 phenotype macrophages and lower M2 phenotype macrophages than those in the carrier protein + RA or Tn vaccine + RA group (Figures 6C,D). Maternal Tn immunization reversed the hyperoxia-induced M1/M2 macrophage polarization to normoxia levels.

## Discussion

Our *in vivo* model revealed that maternal Tn immunization increased maternal and neonatal serum antibody titers and attenuated hyperoxia-induced lung injury in newborn rats, as evidenced by reversing hyperoxia-induced increase in MLI and decrease in vascular density and growth factors. The alleviation of lung injury was associated with a reduction in cytokine and 8-OHdG expression. Therefore, we proposed that maternal Tn immunization attenuates hyperoxia-induced lung injury in neonatal rats through the suppression of oxidative stress and inflammation.

Tn antigen is a pan-carcinoma antigen, expressed on breast, pancreas, colon, lung, and bladder carcinomas, being less common in hematological malignancies (17, 18). Tn is associated with immune disorders in addition to cancers. Tn antigen can be detected on chronic inflammatory tissues in patients with rheumatoid arthritis and osteoarthritis (19). Tn can induce tumor-specific IgG antibodies in mice and in nonhuman primates under appropriate conditions (20). These findings revealed that Tn might be an essential component in the design of humoral-mediated vaccines and suggested that Tn may show immunogenicity and protection in preclinical animal studies. Tn immunization increased serum anti-Tn antibody titers and protected against hyperoxia-induced lung injury in adult mice through the inhibition of NF-κB activity (12). Therefore, maternal Tn immunization may attenuate hyperoxia-induced lung injury in neonatal mice through suppression of inflammation.

Maternal immunization provides protection to the newborns through the transfer of vaccine-induced IgG across the placenta. IgG is the only antibody class that significantly crosses the human placenta. Coder et al. maternally administered radiolabeled humanized IgG2 and found humanized IgG2 in rat embryo/fetal tissues as early as gestation day 11 with a >1,000-fold increase in the amount of total IgG2 by gestation day 21 (21). The concentration of IgG2 in rat embryo/fetal tissues generally remained unchanged from gestation day 11–17 with a slight increase from day 17–21. Moffat et al. compared IgG2X embryonic exposure in rats and found that fetal IgG2X plasma concentrations increased more than six-fold from gestation days 16–21 (22). In this study, we immunized the female rats three times before gestation, on gestation day 7, and at delivery and observed that Tn immunization increased serum anti-Tn antibody titers and protected against hyperoxia-induced lung injury in neonatal rats. These results indicate that maternal immunization is a potential strategy to prevent and treat neonatal diseases. Further studies are needed to determine total IgG and complement factors to elucidate the immunological mechanisms that mediate the beneficial effects of maternal immunization.

Hyperoxia exposure for 7 days increased oxidative stress in the neonatal murine lungs (23, 24). 8-OHdG is a DNA base-modified product generated by reactive oxygen species as a marker of oxidative DNA damage and its levels in target tissues are correlated with other oxidative stress markers (25–27). The expression of 8-OHdG reflects the oxidative stress level in the lung tissues and its expression was elevated in the hyperoxia-exposed neonatal rat lung tissue and primary cultured neonatal rat alveolar epithelial type II cells compared with the normoxic controls (28). Positive signals for 8-OHdG increased in the hyperoxia-exposed rats, and signals were mainly found in the nuclei of epithelial cells. Maternal Tn immunization significantly decreased the hyperoxia-induced increase in the number of positive 8-OHdG cells. These results suggest that anti-Tn antibody-suppressed oxidative stress formation and support that anti-oxidant enzymes are effective in reducing hyperoxia-induced neonatal lung injury (24, 29).

VEGF is a potent endothelial cell mitogen that regulates angiogenesis and alveolar development (30). PDGF is crucial for alveolarization of the normally developing lung (31). We determined VEGF and PDGF-B expression as their mRNA and protein expression was decreased in the hyperoxia-exposed neonatal mice and piglet lungs (32, 33). In this study, we demonstrated that rats in the carrier protein + O_2_ group exhibited significantly decreased VEGF and PDGF-B expression than those in the carrier protein + RA or Tn vaccine + RA group. Maternal Tn immunization significantly reversed the hyperoxia-induced decrease in VEGF and PDGF-B to normoxic levels. These results suggest that maternal Tn immunization enhanced vascular and alveolar development through the induction of growth factors in neonatal rats.

In addition to the traditional host defense, inflammation, and scavenging functions, macrophages have broader functions, including vital roles in tissue repair and organ development (34–36). Animal studies have demonstrated that neonatal hyperoxia exposure increases macrophage infiltration into alveolar airspaces (37, 38). In this study, we used immunohistochemistry to detect the macrophage infiltration on the lung tissue sections. Macrophage phenotype was assessed through immunostaining for iNOS (M1 macrophage marker) and Ym1 (M2 macrophage marker) as hyperoxia increases iNOS expression in macrophages and hyperoxia-exposed murine lungs and inhibits the M2 phenotype in macrophages (39, 40). The rats in the carrier protein + O_2_ group significantly exhibited more M1 macrophages than those in the carrier protein + RA or Tn vaccine + RA group. These results indicate that hyperoxia promotes the M1 phenotype in macrophages and suggest that the M1/M2 polarization mediates the pulmonary effects of hyperoxia in the developing lung. Our study increases the understanding of the role of macrophage polarization in hyperoxia-induced injury to the developing lungs.

In conclusion, we observed that Tn immunization increases serum anti-Tn antibody titers in mothers and neonates, inhibits lung inflammation and oxidative stress, and enhances lung development in the neonatal hyperoxia-exposed rats. These findings indicate that Tn activation may be involved in the mechanism of proinflammatory cytokine release and lung injury and suggest that the Tn vaccine may be a promising treatment modality against hyperoxia-induced lung injury in neonates. Future studies are necessary to evaluate the direct therapeutic effects of anti-Tn antibody on hyperoxia-induced lung injury.

## Ethics Statement

Animal care and experimental procedures were performed in accordance with the guidelines of the Laboratory Animal Care Committee of Taipei Medical University (LAC-2017-0291). Sprague–Dawley rats (6 weeks old) were obtained from BioLASCO Taiwan Co., Ltd and were maintained in a pathogen-free facility and air-conventional animal housing on a 12-h light/dark cycle. The care and housing of experimental animals were approved in accordance with the guidelines of the Laboratory Animal Care Committee of Taipei Medical University.

## Author Contributions

C-MC and JH: designed and performed the experiments; C-MC, JH, and H-CC: analysis and interpretation of data and drafted and approved the manuscript.

### Conflict of Interest Statement

The authors declare that the research was conducted in the absence of any commercial or financial relationships that could be construed as a potential conflict of interest.

## Supplementary Material

Supplement Figure 1 Experimental design of the study timeline and rat treatment groups.
