# Supplementary figures and images for: Maternal Tn Immunization Attenuates Hyperoxia-Induced Lung Injury in Neonatal Rats Through Suppression of Oxidative Stress and Inflammation
Source: Front Immunol. 2019 Apr 4;10:681. doi: 10.3389/fimmu.2019.00681 (PMC6458300; doi:10.3389/fimmu.2019.00681)

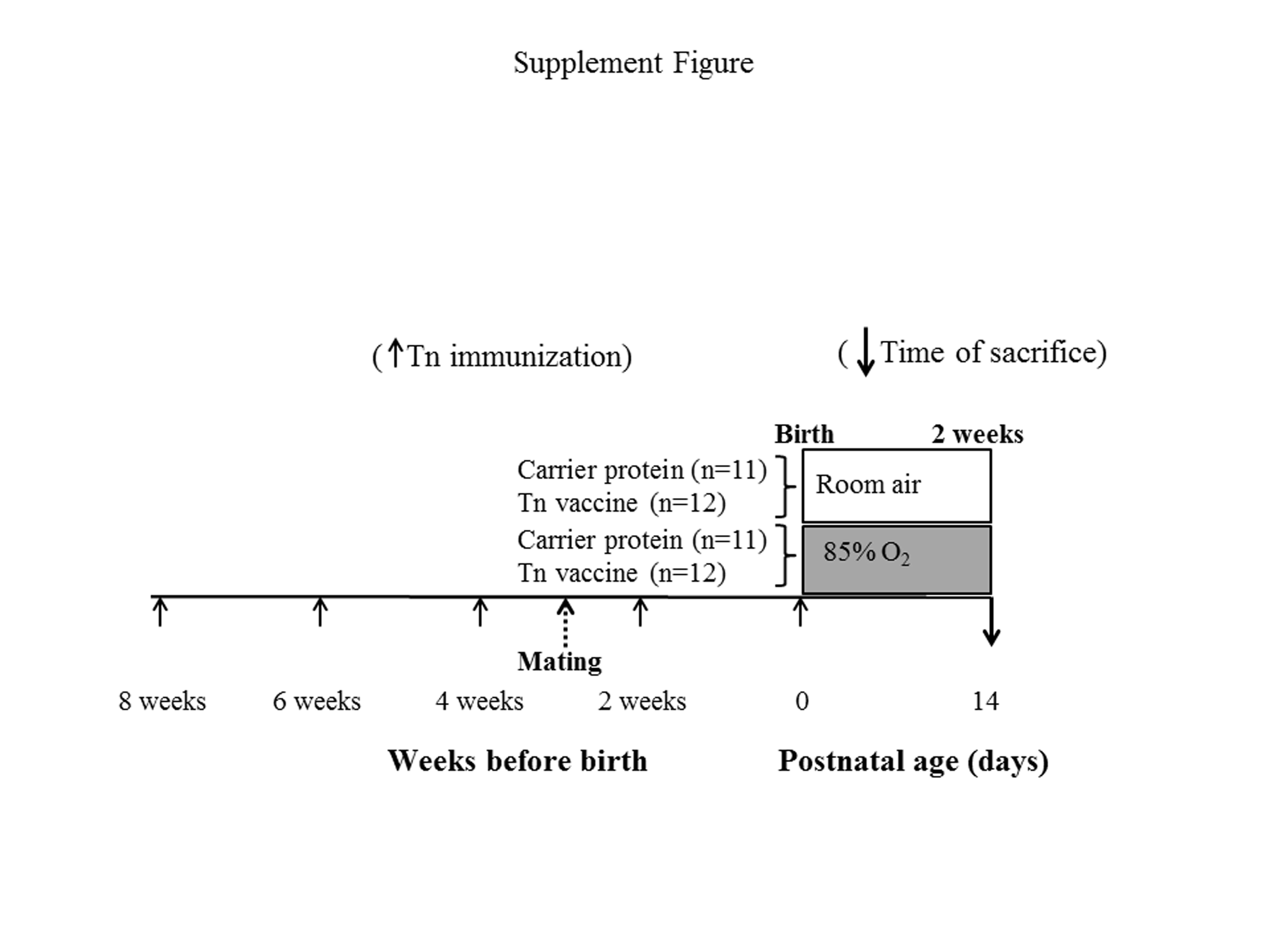

Supplement: Supplement Figure 1 — Experimental design of the study timeline and rat treatment groups. [file Image_1.TIF]
